# Orphan medicinal products in Europe and United States to cover needs of patients with rare diseases: an increased common effort is to be foreseen

**DOI:** 10.1186/s13023-017-0617-1

**Published:** 2017-04-03

**Authors:** Viviana Giannuzzi, Rosa Conte, Annalisa Landi, Serena Antonella Ottomano, Donato Bonifazi, Paola Baiardi, Fedele Bonifazi, Adriana Ceci

**Affiliations:** 1 Fondazione per la Ricerca Farmacologica Gianni Benzi onlus, Via Abate Eustasio, 30 – 70010 Valenzano, Italy; 2 Consorzio per Valutazioni Biologiche e Farmacologiche, Via L. Porta, 14 – 27100 Pavia, Italy; 3 Istituti Clinici Scientifici Maugeri SpA SB, Via Salvatore Maugeri, 4 – 27100 Pavia, Italy

**Keywords:** Orphan drugs, Orphan designations, Medicinal products for rare diseases, Register, Therapeutic needs

## Abstract

**Background:**

In the European Union (EU) and United States (US), specific regulations have been released to provide incentives to develop and sell orphan medicinal products.

We analysed the status of orphan drugs designated that not yet received a marketing authorisation or already marketed for patients affected by rare diseases in the EU and US up to December 2015. For each drug, the following data were extracted: designation date, active substance(s), orphan condition and indication, trade name, approved therapeutic indication, approved ages, genetic nature of disease and if affects children.

**Results:**

In the EU, 1264 Orphan Drug Designations have been granted and 133 medicinal products were approved covering a total of 179 indications and 122 rare conditions. Among these, 79 were approved under Regulation (EC)141/2000 (65 still listed in the Orphan Medicinal Products Register and 14 lost the orphan designation but still authorised) and 23 were approved centrally by the European Agency before the Orphan Regulation entered into force. On the other hand, in the US 3082 designations and 415 orphan products, covering a total of 521 indications and 300 rare conditions, were granted. As a result, the mean of designations per year is 79 in the EU and 93.4 in the US, while the mean of approved indications per year is 8.5 in the EU and 15.8 in the US.

No orphan product is marketed in the EU for bone and connective tissue, ophthalmic, poisoning/overdose, renal, urinary and reproductive rare diseases. Among the marketed medicinal products, only 46.6% in the EU and 35.2% in the US are approved for children.

If all the existing market approvals were merged, 362 additional therapeutic indications in the EU and 72 in the US would be covered.

**Conclusions:**

Our data show that notwithstanding the incentives issued, the number of medicines for rare diseases is still limited, and this is more evident in certain therapeutic areas. However, by merging all the existing approvals, patients would benefit of substantial advantages in both geographic areas. Efforts and cooperation between EU and US seem the only way to speed up the development and marketing of drugs for rare diseases.

## Background

Research and scientific progress in the rare disease field is a challenging objective, since currently only few highly specialised research centres deal with each specific condition. Reasons accounting for this rely on the small number of patients and the scarce economic return for companies. Patients are geographically dispersed, few patients can be recruited in clinical trials, and standard Randomised Controlled Trials (RCTs) are of limited feasibility. This makes longer and more difficult the development process of drugs. In the European Union (EU), United States (US) and elsewhere, specific regulations have been released to provide incentives for companies to develop medicines for diseases with a small market, and the status of “orphan” drug has been created [1, 2]. An “orphan drug” is for the diagnosis, prevention or treatment of a disease ***so rare*** that the *cost* of developing would *not be covered* without additional incentives.

In details, in the EU a medicinal product is designated as “orphan” if it is intended for the diagnosis, prevention or treatment of a life-threatening or chronically/seriously debilitating condition or affecting not more than five in ten thousand persons in the EU or that without incentives it is unlikely that the marketing of the product in the Community would generate sufficient return to justify the necessary investment. In addition, no satisfactory method of diagnosis, prevention or treatment of the condition in question shall have been authorised in the EU or, if such method exists, the medicinal product shall be of significant benefit to those affected by that condition [1].

In the US is orphan any drug intended to treat a disease or condition that affects less than 200.000 persons in the US (corresponding to a prevalence of 7.5 every 10.000 individuals) or affects more than 200.000 people and for which there is no reasonable expectation that the cost of developing and making it available, will recovered from sales [2]. According to the EU and the US regulations, for drugs designated as “orphan”, pharmaceutical companies are entitled to receive incentives including grants, research support, fee waivers/reduction, market exclusivity, and public diffusion of orphan innovation [3], as shown in Table 1, in order to support the availability on the market and to avoid the product is abandoned, or the development is delayed [4].

**Table 1:** Key incentives of the orphan legislation in the EU and US

Incentives	In EU	In US
Marketing exclusivity	10 years + 2 if paediatric	7 years
Clinical development costs	–	tax credits (up 50% of clinical development costs)
Orphan designation	free of charge	free of charge
Support from agency during the development process	free of charge protocol assistance	free of charge OOPD (Office of orphan Products Development) assistance
MAA	40% fee reduction; free of charge for SMEs and for paediatric products	fee reduction
Fee reductions for SMEs	90% of fee reduction for post authorisation inspections; free of charge pre-authorisation inspections, post-authorisation activities, including annual fees, during the first year after marketing authorisation	–
Public funds	(possible) incentives from EC (i.e. research grants)	grants and contract for development of orphan drugs
	(possible) incentives in single Member States for research, development and MA	

Many “orphan” products would not have been developed outside the public funded orphan scheme, as Hudson and coll. demonstrated for enzyme-replacement therapies for the treatment of various mucopolysaccharidoses [5]. On the other hand, as Westermark and Linares pointed out, “patients with rare diseases still face substantial problems and only a minority of their needs have been addressed so far” [6]. In this context, the rarity of pathologies and the geographical dispersion represent hurdles for conducting adequate studies and trials [7], especially because a great part of rare diseases (nearly 50 to 75%) manifest during childhood [8, 9], and paediatric trials have demonstrated to be more and more difficult [10, 11].

Public registries and databases are key tools to increase knowledge on rare diseases and facilitate research [12, 13]. Many registries focused on rare conditions exist, while databases on orphan drugs are few [14]. EuOrphan is a database focused on drugs aimed to diagnose, prevent or treat a rare disease. It was created by Consorzio per Valutazioni Biologiche e Farmacologiche in the framework of a funded European IT-Technology project (eTen 510774 2003/C 118/19), as described by Stakišaitis and coll [15]. Since 2008 it is voluntary managed and regularly updated by Fondazione per la Ricerca Farmacologica Gianni Benzi Onlus [16].

EuOrphan links administrative and scientific data on designated and marketed drugs for rare diseases sourced by official European Commission (EC) [17] and Food and Drug Administration (FDA) [18] databases.

To the aim of this paper, we used data from EuOrphan to depict the status of the orphan drugs designated that not yet received a marketing authorisation or already marketed for patients affected by rare diseases in the EU in comparison with the orphan drugs marketed in the US. We also analysed the advantages resulting for patients, if the Orphan Drug Designations (ODDs) and approvals would be merged between EU and US territories.

## Methods

### Sample

The sample of the analysis included:

#### Designations

Orphan Medicinal Products (OMPs) designated in the EU in accordance with the Orphan Regulation (EC) 141/2000 [1], as listed in the Community Register of Orphan Medicinal Products for human use; orphan drugs designated in the US, as available from the register “FDA Orphan Drug Designations and Approvals” since 1983 [18].

#### Medicinal products approved for a rare condition

To the aim of our research, we considered all the medicines approved for a rare condition in the EU and listed in the EC Pharmaceutical Community Register [17] that includes: 1) OMPs approved for effect of Regulation (EC) 141/2000 [1] by December, 31^st^ 2015; 2) “orphan-like drugs” [19, 20]; 3) non-orphan medicinal products (non-OMPs) approved for a rare condition before the Regulation [1] entered into force; 4) medicinal products withdrawn by the EU OMPs Register but still marketed. Orphan drugs approved by FDA, as listed in the register “FDA Orphan Drug Designations and Approvals” [18].

The period of evaluation was from the entry into force of the orphan legislations in the EU and in the US to December, 31^st^ 2015.

### Collected data

#### Designations

Active substance(s), designation date, sponsor, orphan condition, the genetic nature of disease and if the condition affects children.

#### Medicinal products approved for a rare condition

Trade name, ATC (Anatomical Therapeutic Chemical Classification System) code, approved therapeutic indication, approved ages, approval date, Marketing Authorisation Holder (MAH).

We considered that designations may be awarded to multiple OMPs targeting the same rare disease, but multiple designations may be awarded to one OMP targeting different rare diseases, too, and a single OMP could have more than one indication. In addition, more than one sponsor may obtain a designation for the same active substance and condition.

#### Rare condition

Rare conditions were derived by the orphan designations/approval opinions as published in the official EU and FDA registries. In case of drugs which never received an ODD, the rare condition has been identified in Orphanet [21].

### Data sources

European Medicines Agency (EMA) website (register of designated Orphan Medicinal Products, list of European Public Assessment Reports (EPARs), list of Class Waivers) [22]; European Commission (EC) Community Register of medicinal products [17]; FDA Orphan Drug Designations and Approvals [18]; Orphanet [21], PubMed [23].

### Classifications

For the purpose of the analysis, rare conditions were classified according to the therapeutic area, their genetic origin, and if affect the paediatric population.

Each rare condition was assigned to the following disease areas, set from the existing classifications, such as MedDRA, ICD-10 and Orphanet:

1. Bone and connective tissue diseases
2. Cardiovascular and respiratory diseases
3. Dermatological diseases
4. Endocrine diseases
5. Gastrointestinal and hepatobiliary diseases
6. Haematological diseases
7. Inborn errors of metabolism
8. Infectious and immune system diseases
9. Neurological and psychotic diseases
10. Ophthalmic diseases
11. Oncologic diseases
12. Poisoning/overdose
13. Renal and genitourinary hereditary diseases
14. Others.

The information on the genetic nature of the disease and if the condition affects the paediatric population was searched on Orphanet [21]. Information on diseases affecting adults only was also checked from the EMA list of Class Waivers [22]. If not available, a literature search was performed.

The age groups for which the drug is indicated were classified in accordance with the ICH (International Conference on Harmonization) Topic E11 Guideline [24] and Paediatric Regulation [25]: PRETERM NEWBORN INFANTS: up to 36 weeks gestation; TERM NEWBORN INFANTS: 0–27 days; INFANTS AND TODDLERS: 28 days–23 months; CHILDREN: 2–11 years; ADOLESCENTS: 12–17 years (12–15 years in US); ADULTS: over 18 years (over 16 in US).

### Assumptions

In order to compare and merge the ODDs and drug approvals granted in the EU and US, we assumed that:

- The same designation is available both in the EU and US if the active substance, the rare condition and the MAH are the same between the two territories or they are able to identify one or more sub-contractors for drug distribution worldwide;
- The same medicinal product is available on both the markets if the active substance, the rare condition, the therapeutic indication and the age covered by the indication are the same between the two territories.

With the support of experts, we standardised the names used for active substances and conditions, because we also found several differences between the ODDs obtained by the same sponsor (also for the same active substance and for the same condition).

### Procedures for data quality, integrity and consistency

With the aim to guarantee data quality, integrity and consistency of our database, different steps and responsibilities were foreseen, as described below. A Data Manager (DM) was in charge of extracting data from EMA and FDA online databases every six months and updating the EuOrphan database. Data extracted were cleaned and filtered by in order to implement the database.

When the EuOrphan database is correctly updated, the Scientific Data Manager (SDM) was responsible for implementing all the scientific information such as disease characteristics, therapeutic area, genetic origin of the disease and if affects children.

Finally, the Scientific Reviewer (SR) with the help of experts when needed, performed a final check of the information to be added to the database including standardisation of the names used for active substances, conditions and indications.

**Fig. 1:**
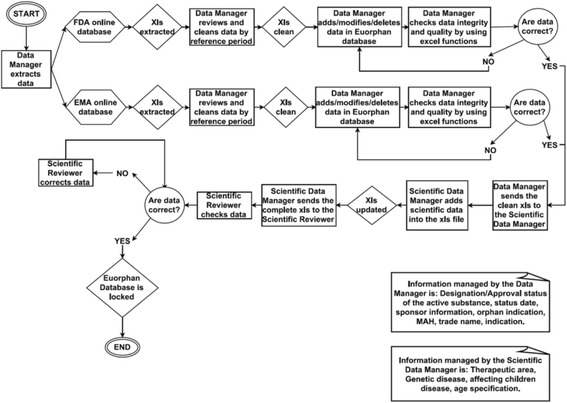
EuOrphan database update – process flowchart

Figure 1 shows in detail the flow of this process.

### Statistical analysis

Descriptive statistics were performed for all the recorded variables. Trends across time of ODDs and marketed products were analysed descriptively and in terms of average figures over time, comparisons between EU and US were performed by means of unpaired Student’s *t* test assuming the robustness of the test for deviation from normality. Differences between occurrences in the number of ODDs or marketed drugs according to different stratification criteria (e.g. genetic diseases, EU vs US) were tested by means of chi-square test. The analyses were performed using SPSS statistical software.

## Results

### Orphan designations

By December 31^st^ 2015, 992 active substances from the EU OMPs Register, corresponding to 1.264 ODDs, and 2.270 active substances, corresponding to 3.082 ODDs, from the “FDA Orphan Drug Designations and Approvals” register were entered into EuOrphan database. The average number of designations per active substance resulted to be slightly higher in the US compared to the EU (1.36 vs 1.27).

**Fig. 2:**
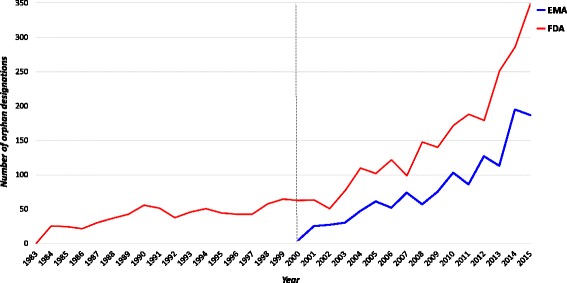
Orphan designations in the EU and US released per year

Figure 2 shows the trends in the period 1983–2015 for ODDs released per year by the EMA and the FDA. On average, across the years 2000 and 2015 where data are available for both the Agencies, a significantly lower number of ODDs is observed for EMA compared to FDA (79 vs 93.4, *p* = 0.009).

In the EU, the ODDs covered 370 rare diseases, of which 319 affect children (86.2%) and 161 are genetic (43.5%). In the US, the ODDs covered 800 rare diseases, of which 672 affect children (84%) and 296 are genetic (37%). Therefore, the most of rare diseases covered by an ODD affect children both in the EU and US (n.s. *p* = 0.34), while the percentage of genetic diseases covered by an ODD is higher in the EU compared to the US (*p* < 0.001).

**Fig. 3:**
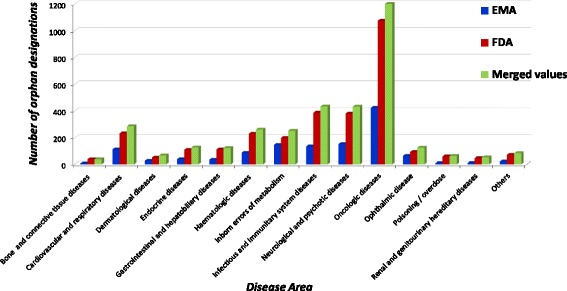
Distribution of orphan designations per disease area

Regarding the disease area, the largest number of ODDs was detected in the oncologic area for both EU and US, as shown in Fig. 3. Other ODDs have been granted as supportive therapies to oncologic diseases (10 in the EU and 64 in the US) and classified in different therapeutic areas (data not shown).

If all the existing EU and US designations were merged between EU and US, a greater number of active substances designated as “orphan” would be potentially available to patients both from the EU and US. Accordingly, we calculated the total number of ODDs obtained by merging all the existing EU and US designations (Fig. 3, green column). Data shown in Fig. 3 demonstrate that the merge of the ODDs globally granted in the EU and US resulted in 3552 ODDs. These designations would cover 1015 rare diseases.

On average, the number of ODDs that reached the market approval was globally low, but in the EU this figure is significantly lower (*P* < 0.001) compared to the US (8.5 versus 15.8).

Furthermore, the highest number of ODDs was sponsored by all commercial entities, big pharma or regulatory consultancy agencies both in the EU and US but not the same firms are the mostly represented in the two contexts.

### Marketed drugs

In the EU, a total of 133 medicinal products, covering 179 indications for rare diseases, resulted on the market. Among these approved indications, 139 were approved by the European Medicines Agency under the EU Regulation (EC)141/2000 [1] (116 of them are still listed in the EU OMPs Register while 14 lost the ODD but are still present on the market), 40 were approved centrally by the European Medicines Agency before the Orphan Regulation [1] entered into force (6 of them were classified by the Agency as “orphan-like drugs”).

In the US, 415 orphan drugs (covering 521 orphan indications) were approved. The average number of indications per marketed medicinal product is slightly higher in the EU compared to the US (1.34 vs 1.25).

**Fig. 4:**
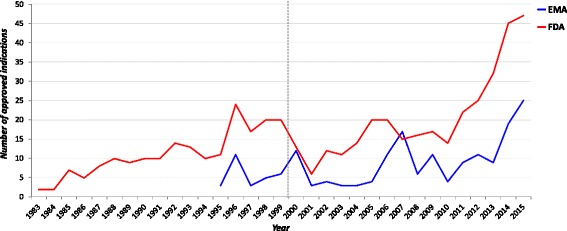
Distribution of indications for rare conditions per year

Figure 4 shows the distribution of indications for rare conditions in the EU and US per year. Similarly, to ODDs, the mean number of marketed products per year in the EU is significantly lower than the number of marketed product per year in the US (8.5 vs 15.8, *P* < 0.001).

In addition, the percentage of genetic diseases covered by an approved OMP is higher in the EU compared to the US (47.5% vs 40.7%, *p* = 0.002): in the EU, 122 rare conditions are covered by an authorised indication, of which 58 are genetic; in the US, 300 rare conditions are covered by an authorised indication, of which 122 are genetic.

**Fig. 5:**
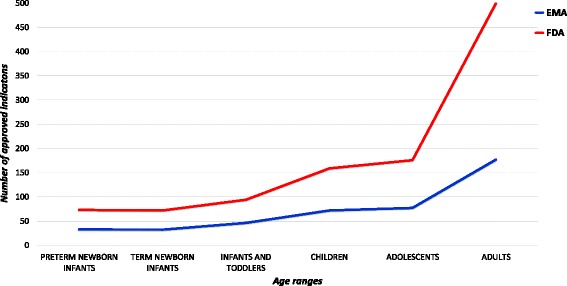
Distribution of indications in the EU and US per age groups. Legend: label not available for 7 ODDs

Distributing the approved indications by age, as stated in the authorised product information leaflet (Fig. 5), we demonstrated that 62 and 161 medicinal products, covering a total of 77 and 186 paediatric indications, were approved for the whole or part of the paediatric population in the EU and US respectively. Noteworthy, the official label was not available for seven ODs in the FDA database. This means that 43% in the EU and 35.7% in the US of indications were approved for children.

We also calculated the number of indications approved only for adults even if the condition affects children. This number resulted 64 in the EU and 242 in the US. This means that 64/140 (45.7%) in the EU and 242/343 (70.5%) in the US (*p* < 0.0001) of the indications for conditions affecting children are not approved for the paediatric population.

For younger children, in the EU 33 and 32 out of the total number of indications (18.4% and 17.9%) were approved for preterm and term newborn infants respectively; in the US, even if the total of the indications approved for preterm and term newborn infants were about twice higher than in the EU (73 and 72 respectively), the percentages are lower for both preterm and term newborns (14% and 13.8% respectively).

**Fig. 6:**
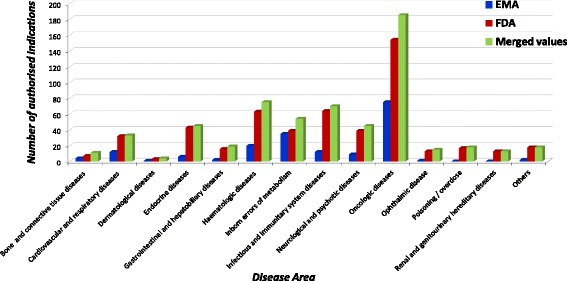
Distribution of authorised indications for rare conditions per disease area

In line with designations, the largest number of medicinal products was detected in the oncologic area for both agencies as shown in Fig. 6. However, in Europe no drug was marketed for renal, urinary and reproductive rare diseases and “poisoning/overdose”.

The MAHs with the highest number of designations approved for the market are all commercial entities, big pharma or regulatory consultancy agencies.

As for the designations, considering the number of therapeutic indications approved for the market, a total of 348 rare diseases would have a marketed drug in almost all the disease areas, as detailed in Fig. 6 (green column): 362 additional therapeutic indications in the EU and 72 in the US would be covered.

## Discussion

In line with previous publications [6, 26–29], the results deriving from this study performed on EuOrphan data, reveal that, thanks to the incentives issued by the regulations aimed at encouraging the development of OMPs, the number of medicines for rare diseases greatly improved. Actually, these regulations have clearly stimulated the development of drugs for rare conditions, even those previously untreatable, as demonstrated in the EU [30].

Up to 2015, in the EU and US ODDs result 1264 and 3082 respectively and the number of drugs approved for the market 133 (10.5% out of the total of ODDs) in the EU and 415 in the US (13.5% out of the total of ODDs). This is in line with previous data claiming that the US continues to have the most designations and the most approvals [31], as well as with the reported “success rates” (the proportion of orphan medicines that receive marketing approval after receiving an orphan designation) resulting similar in the EU and US [32].

This numerical disparity might be partially correlated with the different date of entering into force of regulations stimulating the development of orphan drugs for rare diseases, since in the EU the orphan legislation [1] came into force 20 years after the Orphan Drug Act in the US [2].

Many publications dealing with the legislative frameworks and differences among them are available in the most relevant literature [31–33]. Firstly, we should take into account that the legislations and policies encouraging the development of these medicines are to some extent similar but not the same. The eligibilities for the ODD slightly differ depending on the legislation and policies adopted by each region [31]. For example, the definition of “rare disease” based on prevalence is not universal and depends on the legislation and policies adopted by each region or country [34]: in the EU, a rare disease is a condition affecting less than 5 individuals in 10.000 people, while in the US a rare disease is a condition affecting less than 7.5 in 10.000 individuals.

In the EU, alternatively to the prevalence of the condition, the second criteria to designate an OMP considers the lack of sufficient return generated from the marketing of the medicinal product intended for a life-threatening, seriously debilitating/serious and chronic conditions (even when the prevalence is higher). Interestingly, in the EU only one drug received the ODD on the basis of this second criteria not referring to the low prevalence, namely the “Recombinant modified vaccinia virus Ankara expressing tuberculosis antigen 85A” for the prevention of tuberculosis disease in Bacille Calmette-Guérin (BCG) vaccinated individuals [35]. Secondly, the differences between incentives set by the two legislations issued in the US or in EU to stimulate the interest of pharmaceutical companies in investing in the orphan drug sector (the Orphan Drug Act and European Regulation 141/2000) [1] might be carefully considered. Grants, research design support, fee waivers, tax incentives, orphan drug market exclusivity, and public diffusion of orphan innovation are main incentives for orphan R&D (Research and Development) [4]. Both in the EU and US, the ODD and the protocol assistance are free of charge. Protocol assistance is a procedure through which regulatory authorities provide companies developing OMPs scientific advice on the type of studies to be carried out to demonstrate the quality, efficacy, safety. In the EU, the protocol assistance or scientific advice is given by the EMA Committee for Medicinal Products for Human Use (CHMP) on the recommendation of the Scientific Advice Working Party (SAWP). In the US, it is given by the FDA Office of Orphan Products Development (OOPD). Parallel scientific advice/protocol assistance between EMA and FDA is available. In addition, the allocation of public funds and fee reduction are available in both Agencies. On the other hand, the granted marketing exclusivity is different (7 years in the US and 10 years in the EU). What makes really the difference is the economic support for the clinical development costs, which is not set out by the orphan legislation in the EU, while in the US there are tax credits (up 50% of clinical development costs). However, EC research programmes, such as the Sixth and Seventh Framework Programmes and the ongoing Horizon 2020, have granted and are granting funding also for OMPs development and additional funds are requested to be provided by each Member State.

Our analysis, which considered not only OMPs but also the other medicinal products centrally approved in the EU for a rare condition, highlights that the numerical difference between EU and US is reduced if we consider non-OMPs marketed in the EU before the entry into force of the Regulation in the EU [1]. Approved OMPs are not the only medicines available on the market. The title of “orphan” drug is gained if the sponsor aims to receive incentives for the development of an active substance intended for a rare disease (according to the orphan legislation).

Our data confirm the most significant role of pharmaceutical companies/profit sponsors in the development of orphan drugs.

If we look at specific therapeutic needs, we confirm that the oncologic area is the most represented one, because it includes the highest number of ODDs and the highest number of approved medicinal products both in the EU (377 and 32 respectively) and in the US (917 and 121 respectively), in line with previous data [14, 36]. A significant number of ODDs has been developed for the treatment of cystic fibrosis, amyotrophic lateral sclerosis in both countries, as well as Duchenne muscular dystrophy and graft versus host disease in the EU.

Moreover, data from this study reveal that a great part of genetic rare diseases has still an unmet therapeutic need. Furthermore, all the 64 ODDs referring to the following rare diseases did not receive the marketing approval either in the EU (34 ODDs) or US (30 ODDs): retinitis pigmentosa, corneal graft rejection, Leber's congenital amaurosis, macular telangiectasia, focal segmental glomerulosclerosis, low-flow priapism, nephrotic syndrome, primary membranoproliferative glomerulonephritis, uremic pruritus, autosomal dominant polycystic kidney disease. We also demonstrated that in the EU no drugs are marketed for poisoning/overdose, renal, urinary and reproductive rare diseases.

With regards to paediatric medicines, our data demonstrated that despite of the interest and the need for drugs approved for children, about half of drugs approved in the EU and US for a rare disease affecting children was not granted a paediatric indication. Interestingly, the EU has a greater percentage of drugs with a paediatric indication for rare conditions affecting children.

Furthermore, we found that few drugs were approved for younger children, such as neonates (about 18% in the EU and 14% in the US). So, in the US, even if the total of the indications approved for preterm and term newborn infants was about twice higher than in the EU, the percentage out of the total number of approved indications is lower.

More in depth analyses are necessary on the paediatric interest of each OMP to better evaluate the paediatric needs for conditions affecting children.

So far, great efforts have been made by the European Medicines Agency on the availability of drugs for rare diseases, and continued efforts are still required from the EU, its institutions and Member States [30]. As an example, the Agency is going to shortly launch the Priority Medicines (PRIME) scheme to push on the development of medicines with unmet medical needs.

Finally, we tried to calculate the total coverage of drugs marketed for rare diseases in the EU and US if the efforts of the two agencies were joined and the designations and approvals were merged between the two territories. In this case, patients affected by rare diseases would benefit of a greater number of drugs in all the disease areas, both in the EU and US.

Similarly, Murakami and Narukawa demonstrated that if the EU, US and Japan joined their ODDs, out of about 5000 designations, approximately 800 designations were common among the USA, EU, and/or Japan [31]. However, as we experienced in performing this analysis, several difficulties may arise in merging designations and approvals, because terms and classifications are not standardised and sponsors may differ between the two regions as well as may differ from the manufacturer/patent holder.

This joint effort would be made possible if regulatory procedures were harmonised, and through efforts and cooperation between the territories. The EMA and the FDA started working together in 2008 and a Common EMA/FDA Application Form for ODDs has been set up for sponsors seeking orphan drug status in both the US and EU. The FDA and the EMA have also agreed to accept the submission of a single annual report from sponsors of orphan products (drugs and biologics) designated for both the US and EU. The parallel submission of orphan designation applications has been very successful, with 62% of dossiers submitted in parallel to the EMA and the FDA in 2012. To further encourage applications for orphan designation to be submitted in parallel by EMA and FDA, the two agencies also provide parallel scientific advice to sponsors during the development phase of their products [37].

The expectations of both the agencies with respect to the data are near about similar and sponsors can have a common strategy at the very early stages of product development [26]. Actually, this example of a common approach seems the best way to go ahead.

Notwithstanding the giant steps made from the European and American Agencies to harmonize their strategic plans in the field of orphan drugs, we found difficulties in collate information between the two Regulatory Bodies (FDA and EMA), because the terminology and processes are not completely harmonised. We even found different terms of active substance and/or rare condition in ODDs obtained in the EU and US by the same sponsor. Therefore, we were often unable to uniform terms and to consider different two ODDs which were practically the same.

## Conclusions

Our results suggest that more efforts seem necessary to increase the number of drugs obtaining the Marketing Authorisation, which represents the first step for the availability of medicines on the market, thus increasing the coverage of patient needs. A more integrated approach between Europe and United States in terms of shared decisions, approvals, etc. would surely speed up the development and then the marketing of drugs for rare diseases. But we are aware that this is a challenging goal.

Regarding the use of systematic collection and stored of valid information on ODDs we consider that our database demonstrated to be a useful tool to increase knowledge on rare diseases and facilitate research. In particular, EuOrphan has been recognised a valid source of information in the context of an EU Commission funded projects on rare diseases, such as InNerMeD-I-NETWORK (Inherited NeuroMetabolic Diseases Information Network, 2012 12 12, Second Health Programme) focused on inherited neurometabolic diseases, and DEEP (DEferiprone Evaluation in Paediatrics - 261483 - FP7-HEALTH-F4-2010).

For the future, we aim to implement the database with data on non-OMPs, namely the medicines approved for rare diseases by the national Agencies and medicines never receiving an ODD while approved by EMA after the entry into force of Regulation (EC) 141/2000 for rare diseases.

Currently, our major commitment is to make the actual database easily accessible for researchers, companies as well as for patients to search for information and to disseminate all the available data on OMPs approved and designated.

## Abbreviations

ATC: Anatomical Therapeutic Chemical Classification System
BCG: Bacille Calmette-Guérin
DM: Data Manager
EC: European Commission
EMA: European Medicines Agency
EPAR: European Public Assessment Report
EU: European Union
FDA: Food and Drug Administration
ICH: International Conference on Harmonization
MAH: Marketing Authorisation Holder
ODD: Orphan Drug Designation
OMP: Orphan Medicinal Product
R&D: Research and Development
RCT: Randomised Controlled Trial
SDM: Scientific Data Manager
SR: Scientific Reviewer
US: United States
